# Integrated Digital and Conventional Treatment Workflow in Guided Complete Mouth Implant Rehabilitation: A Clinical Case Report

**DOI:** 10.3390/dj7040100

**Published:** 2019-10-01

**Authors:** Jason D. Lee, Soomin Jung, Chin-Wei Wang, Sang J. Lee

**Affiliations:** 1 Department of Restorative Dentistry and Biomaterials Sciences, Harvard School of Dental Medicine, Boston, MA 02115, USA; Jason_Lee@hsdm.harvard.edu (J.D.L.); soominjung@gmail.com (S.J.); 2 Department of Health Policy and Management, Seoul National University College of Medicine, Seoul 08826, Korea; 3 Department of Oral Medicine, Infection, and Immunity, Harvard School of Dental Medicine, Boston, MA 02115, USA; jeffwa@umich.edu; 4 Department of Periodontics and Oral Medicine, University of Michigan School of Dentistry, Ann Arbor, MI 48109, USA

**Keywords:** Dental implant, full mouth rehabilitation, CAD/CAM, guided implant surgery

## Abstract

**Clinical Implications:**

The integration of both conventional and digital techniques in the treatment workflow can have a synergistic effect that facilitates the treatment of complex complete mouth prosthetic rehabilitations.

**Abstract:**

The introduction of digital dentistry and CAD/CAM technology has redefined treatment concepts in implant dentistry—computer guided implant placement has become routine practice, and CAD/CAM prostheses are now commonplace. These advances in treatment options and modalities has led to a paradigm shift in the workflow of surgical and restorative treatments. This case report presents a customized staged treatment protocol that involves the strategic retention of teeth to serve as transitional abutments, which will support a computer guided implant surgical guide as well as a fixed interim prosthesis. The treatment protocol also describes an integrated digital and conventional workflow for full mouth implant-supported fixed prosthetic rehabilitations to provide improved patient care with more predictable outcomes and fewer complications.

## 1. Introduction

The challenges involved in the treatment of completely edentulous patients with fixed implant rehabilitations have been reported in the literature [1,2,3,4]. Inadequate surgical and restorative planning can result in both biological and prosthetic failures. A thorough analysis of the available prosthetic space, as well as an evaluation of the functional and esthetic parameters through a diagnostic wax up are critical steps in planning the position and angulation of implants. Advances in three-dimensional (3D) imaging technology and digital scanners have allowed for better visualization of anatomic structures and improved precision and accuracy for implant placement [5,6,7,8]. By merging the DICOM data from CT scans of edentulous arches and STL files from a digital scan of a diagnostic prototype, implant placement can be virtually planned in relation to the final prosthesis in 3D software [9,10]. A surgical template is then fabricated according to the virtual plan by the placement of surgical guide sleeves [11]. The clinical viability of using computer-guided surgery has been investigated and has become a routine practice in implant dentistry [12]. The advantages of a computer guided surgical protocol are manifold: It serves as a tool for preoperative analysis, provides for predictable implant positioning, allows optimal utilization of existing bone with minimum bone augmentation, and facilitates the possibility of a flapless approach [13,14].

Once the implants are placed in prosthetically idealized positions based on the guided surgical planning, the restorative phase is greatly simplified as the appropriate prosthetic space as well as implant access hole locations have been virtually pre-verified. The combination of CAD-CAM technology with computer guided implant surgery not only simplifies the treatment protocol, but can also enhance the final prosthetic outcome [15,16].

The transitional period from a provisional to a definitive prosthesis during a complete mouth implant reconstruction presents a challenge for clinicians and patients, alike. Unexpected procedural complexities may hamper the clinician’s progress, and patients may experience difficulty in accepting and adapting to prosthetic changes. Various techniques have been applied to facilitate this transition including the use of removable dentures and provisional mini-implants, as well as immediate implant loading protocols to reduce the treatment duration and enhance the patient experience [17,18,19,20,21,22]. A staged approach, in which questionable or hopeless dentition are retained as transitional abutments, has been considered beneficial for easing the transition from the provisional stage to the final restoration. This approach involves strategic extractions, implant placement, tooth-supported fixed interim prostheses, and strategic timing of implant loading and restoration [23,24,25]. One major advantage of employing a staged approach is the presence of a fixed provisional throughout the entire treatment period; hence, implants are protected from premature loading and patient acceptance is increased [26,27]. An additional clinical benefit of a staged approach is that the abutments can serve to support both the radiographic and surgical templates in a stable and repeatable position—a simpler alternative to the traditional horizontal fixation pins used to stabilize soft/hard tissue-borne templates.

The aim of this clinical report is to describe an integrated digital and conventional treatment workflow in the management of a fixed implant-supported complete mouth rehabilitation. A staged treatment approach was utilized in conjunction with computer guided implant placement, and definitive prostheses were fabricated using CAD/CAM titanium abutments and monolithic zirconia prostheses. Before starting the treatment, the subject gave her informed consent that records of the case would be available for teaching purposes, including scientific publication.

## 2. Case Presentation

### 2.1. Diagnostic Phase

**Figure 1:**
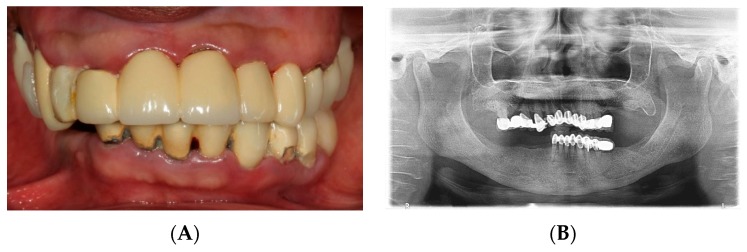
Initial examination. (**A**) Clinical presentation. (**B**) Panoramic radiograph.

A 77-year-old female patient presented to the Advanced Graduate Education Prosthodontics clinic at Harvard Dental Center with the chief complaint of “I would like to chew and smile.” The patient requested fixed restorations and expressed a desire to avoid wearing removable prostheses during her treatment. The patient’s medical history was non-contributory to any dental treatment with normal vital signs and a reported allergy to penicillin. The initial comprehensive clinical (Figure 1A) and radiographic (Figure 1B) examination revealed partial edentulism with defective restorations, inadequate occlusal plane and loss of unilateral posterior support. The patient’s remaining teeth presented with poor to guarded prognoses involving advanced periodontal disease, caries, and Miller’s class III mobility. 

Following a discussion of the benefits, risks and alternative treatment options, the patient consented to maxillary and mandibular fixed implant-supported rehabilitation. Based on the patient’s existing anatomy, the planned prosthetic design, and biomechanical prosthodontic principles, eight implants were planned in the maxillary arch, and six were planned in the mandibular arch. The treatment goals were to restore form, function, and esthetics while maintaining the patient in fixed prostheses throughout the entire treatment period. 

### 2.2. Foundational Phase: Elimination of Active Disease, Extraction and Fixed Provisional Prosthesis

**Figure 2:**
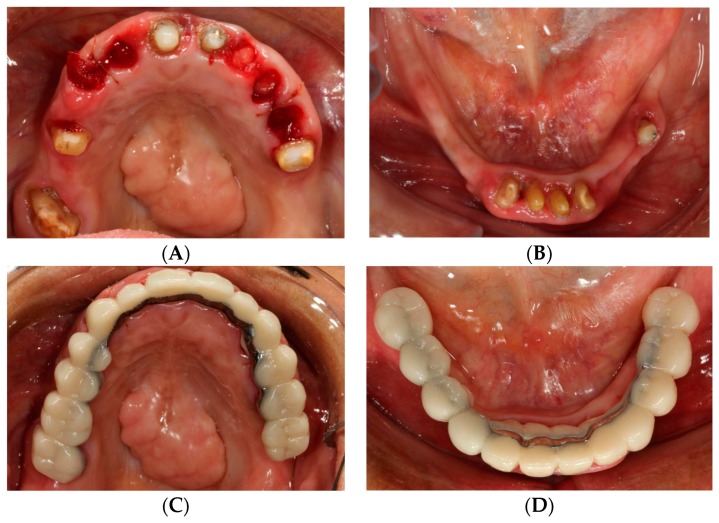
Strategic extraction and tooth-supported fixed provisional prosthesis. (**A**) Maxillary view after strategic extraction. (**B**) Mandibular view. (**C**) Maxillary view with tooth-supported fixed provisional prosthesis. (**D**) Mandibular view.

The first phase of treatment consisted of oral hygiene instruction and elimination of active disease, strategic extraction of teeth and fixed provisionalization. Eight implants were planned in the maxilla at the positions of the lateral incisors, canines, first premolars and first molars, and six implants were planned in the mandible in the areas of the canines, first premolars and first molars. Extraction sites were determined based on the planned implant positions while the remaining abutment teeth were kept to support a fixed provisional as well as the computer guided surgical template during the implant surgical phase (Figure 2A,B). Following the strategic extractions, lab-processed tooth-supported fixed provisional prostheses (Figure 2C,D) were placed and relined with auto-polymerizing acrylic resin (Alike; GC America Inc, Alsip, IL, USA). 

### 2.3. Surgical Phase: Virtual Planning of Implant Placement and Computer Guided Surgery

**Figure 3:**
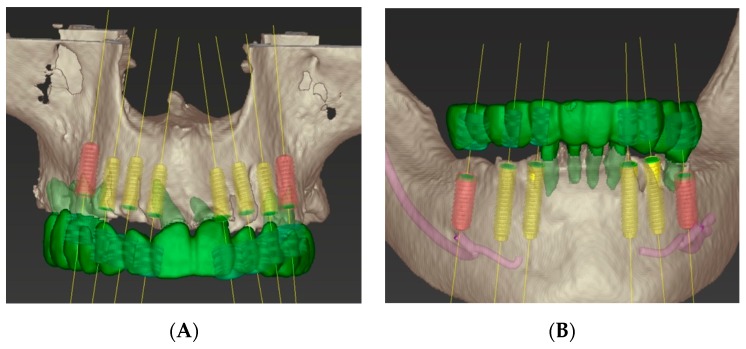
Computer-guided virtual planning. (**A**) Maxillary arch. (**B**) Mandibular arch.

**Figure 4:**
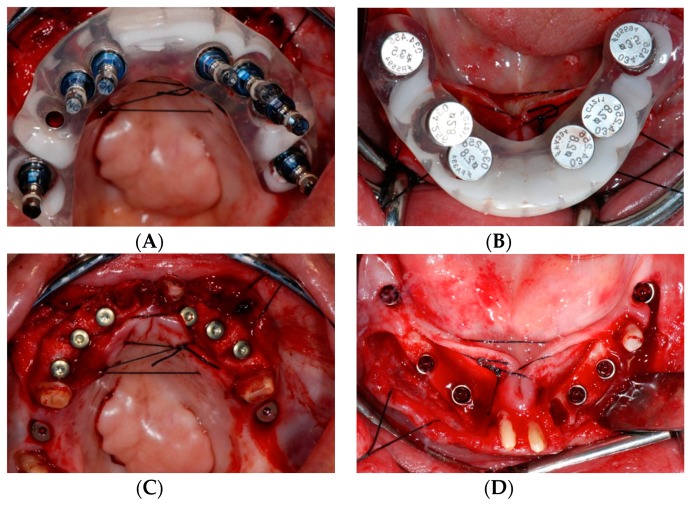
Computer-guided implant surgery. (**A**) Maxillary surgical template with guided mounts. (**B**) Mandibular surgical template with fixation pins. (**C**) Implants placed in the maxilla following guided surgical protocols. (**D**) Mandibular view.

The second phase of treatment involved an integrated digital and conventional workflow for guided implant surgical intervention. Eight weeks after the extractions, radiographic guides were fabricated by duplicating the provisional restorations in a radiopaque acrylic and affixing them to templiX reference plates (Straumann USA, LLC, Andover, MA, USA). Computerized tomography (CT) scans were taken with the patient wearing the radiographic guides stabilized on the transitional abutments. The implant positions were virtually planned in relation to the hard and soft tissue architecture and the planned prostheses (Figure 3A,B) with virtual implant planning software (coDiagnostiX. Straumann USA, LLC, Andover, MA, USA). Based on the virtual planning, the radiographic guides were converted into surgical templates (Straumann USA, LLC, Andover, MA, USA). During the pre-operative visit, the accurate seating of the surgical templates on the transitional abutment teeth was confirmed and irrigation holes were made. The implants were placed as planned following the manufacturer’s recommended surgical protocol and guided surgery instrumentation (Straumann USA, LLC, Andover, MA, USA) (Figure 4A–D). 

### 2.4. Restorative Phase: Second Strategic Extraction and Conversion of Provisional Prostheses with Integrated Digital Workflow

**Figure 5:**
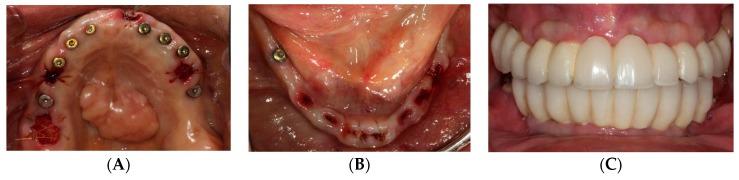
Second stage strategic extraction and conversion of fixed provisional prosthesis. Extraction of remaining abutment teeth. (**A**) Maxillary view. (**B**) Mandibular view. (**C**) Converted implant-supported provisional prosthesis.

After a three-month healing period, the transitional teeth abutments were extracted (Figure 5A,B). The tooth-supported provisional restorations were converted into screw-retained implant-supported provisional prostheses and the soft tissue around the pontic areas were contoured with an ovate pontic design (Figure 5C).

**Figure 6:**
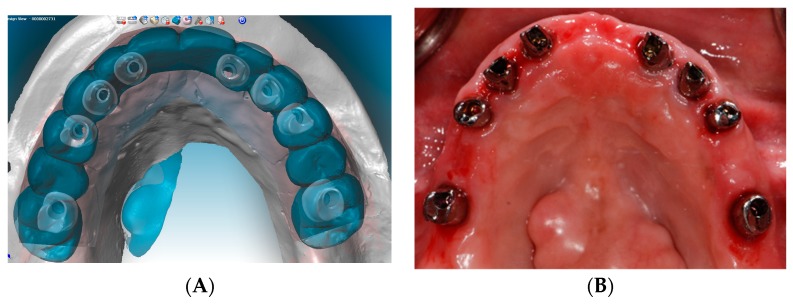

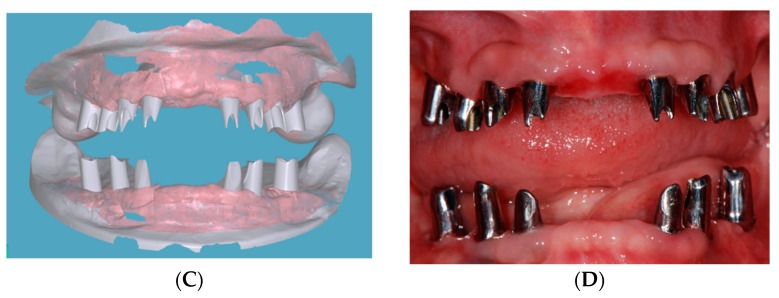
CAD/CAM custom titanium abutments. (**A**) Virtual design of abutments with scanned wax up of definitive prosthesis. (**B**) Intraoral try-in of CAD/CAM custom titanium abutments. (**C**) Virtual design of abutments. (**D**) Intraoral try-in of CAD/CAM custom titanium abutments.

**Figure 7:**
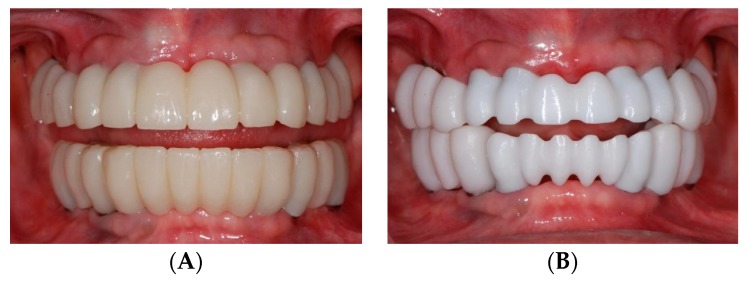
Verification of prototypes and milling of zirconia after re-scanning. (**A**) PMMA prototype. (**B**) Anterior milled zirconia framework and posterior monolithic zirconia.

**Figure 8:**
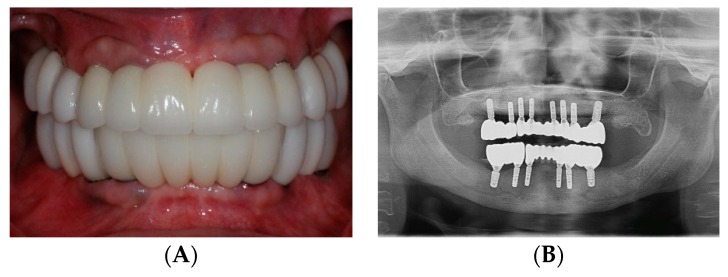
Definitive prosthesis. (**A**) Intraoral view. (**B**) Panoramic radiograph.

During the definitive restorative phase, maxillary and mandibular implant level impressions were made with silicone impression material (Aquasil, Dentsply International Inc., York, PA, USA) using an open tray impression technique. The master casts and the existing provisional casts were scanned and superimposed, allowing for the virtual designing of the abutments and zirconia prostheses (Figure 6A,C). CAD/CAM titanium abutments were fabricated for all the implants (Titanium grade 4, CARES Abutments, Straumann USA, LLC, Andover, MA, USA) (Figure 6B,D). Full contour prototypes (ZCAD Temp Fix, Harvest Dental, Brea, CA, USA) were duplicated and milled based on the scan of the existing provisional restorations. The prototypes were used to evaluate marginal fit and passivity, as well as to verify proper occlusion. Occlusal interferences were eliminated to achieve mutually protected occlusion. Gingival embrasures and pontic areas were adjusted to ensure adequate accessibility and contours for cleansability. (Figure 7A). The verified prototypes were re-scanned and served as a blueprint for fabrication of the definitive anterior zirconia frameworks (Katana Zirconia, Kuraray Noritake Dental Inc. Miyoshi, Aichi, Japan) as well as the posterior full contour monolithic zirconia restorations (Katana Zirconia HT, Kuraray Noritake Dental Inc. Miyoshi, Aichi, Japan). Once fabricated, the anterior zirconia frameworks and posterior full contour crowns were clinically and radiographically evaluated to confirm acceptable fit (Figure 7B). The anterior zirconia frameworks were veneered with porcelain (Cerabien ZR (CZR), Kuraray Noritake Dental Inc. Miyoshi, Aichi, Japan). The segmented definitive prostheses were delivered with self-adhesive universal resin cement (Rely X Unicem, 3M ESPE, St. Paul, MN, USA) (Figure 8A,B). The patient was educated in proper hygiene protocols for the final prostheses, and an occlusal appliance was delivered to minimize attritive wear of the prostheses.

## 3. Discussion

In this case, the transition from failing dentition to a fixed implant-supported complete rehabilitation was accomplished in several phases: Strategic extraction, virtual implant planning, computer guided surgery, and CAD/CAM prosthesis fabrication. Despite advances in the computer guided surgical technology, inaccuracies can accumulate from all stages of the workflow—from CT data acquisition, and data manipulation, to imprecision in the seating of the surgical template. Horizontal fixation pins are commonly used in computer guided implant systems treating edentulous arches. In such cases, however, the reproducible seating of the radiographic guide and surgical template can be a challenge due to the nature of mucosa-borne guided surgery. In the present case, the utilization of transitional tooth abutments allowed for reproducible and accurate positioning of the radiographic and surgical guides. Another benefit of strategically keeping transitional abutments is that quadrilateral positioning of transitional tooth abutments allows for fixed provisional prostheses and uninterrupted healing of the implants. 

In the final restorative phase, the integration of digital technology with conventional prosthodontic techniques allowed for a customized treatment protocol. The use of a digital impression system for a single implant restoration has been found to be more efficient than conventional impression techniques [28]. However, a conventional impression technique was utilized in this case because the accuracy and precision of intraoral digital scanning for full arch fixture level impressions has not been demonstrated. CAD/CAM technology was utilized to design the abutments and fabricate the final prostheses. Virtual superimposition and duplication of an idealized prototype facilitates predictable outcomes by improving prosthesis accuracy, ensuring passive fit, and transferring a verified occlusal scheme from the provisional stage to the final restorative phase. 

**Figure 9:**
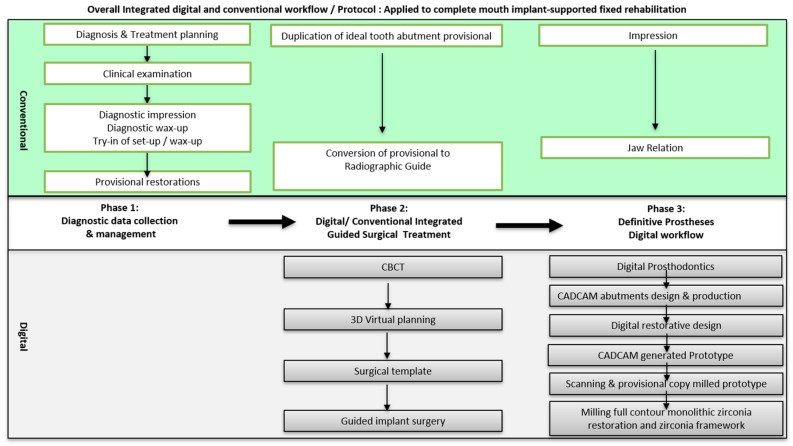
Overall workflow of integrated digital and conventional protocol applied to full mouth implant-supported fixed rehabilitation.

With the introduction of novel digital technology into the field of dentistry, it is necessary to determine the ideal combination of a digital and conventional workflow based on the stages of treatment. In our proposed protocol, the overall workflow can be largely divided into three phases consisting of integrated conventional and digital techniques. Phase 1 is a diagnostic and preparatory phase wherein conventional techniques are used to set the foundation for future surgical and restorative interventions. The end result of this phase is an idealized provisional restoration on transitional tooth abutments. Phase 2 consists of a conventional conversion of the provisional to a radiographic stent, and a digital planning and subsequent guided placement of the implant fixtures. Finally, in phase 3, a conventional technique is utilized for the final impressions of the implants, while a predominantly digital protocol is utilized to arrive at the final prostheses (Figure 9).

A systematic review of implant supported fixed prostheses reported complications such as the fracture of resin teeth or frameworks, material wear, and veneer chipping or fracture [29]. The use of monolithic zirconia eliminates the problem of veneer chipping and reduces the risk of fracture [30,31]. The wear properties of CAD/CAM monolithic zirconia materials have proven to be more favorable than those of feldspathic veneering porcelain, which has been traditionally used in dentistry [32,33]. However, chairside adjustments with diamond burs may introduce microfractures and roughen the surface of zirconia restorations [34,35]. In the present case report, CAD/CAM technology was utilized in the duplication of an idealized acrylic prototype into the definitive zirconia restorations, which minimized chair-side adjustments of the final prostheses and allowed a direct transfer of the verified occlusal scheme developed in the provisional phase.

The overall expected prognosis of the presented treatment is considered good with patient compliance in the wearing of an occlusal guard and following a proper recall and maintenance regimen.

## 4. Conclusions

With adequate planning and proper execution, a phased computer-guided implant surgical protocol and CAD/CAM technology can bring accuracy, efficiency, and predictability to complex full-mouth implant rehabilitation cases. Understanding the limitations of conventional and digital dentistry will allow the clinician to create a synergistic integration of processes from both realms to optimize the management of complex and challenging treatments.

## References

[1] Adell R., Eriksson B., Lekholm U., Branemark P.I., Jemt T. (1990). Long-term follow-up study of osseointegrated implants in the treatment of totally edentulous jaws. Int. J. Oral Maxillofac. Implants.

[2] Zarb G.A., Schmitt A. (1990). The longitudinal clinical effectiveness of osseointegrated dental implants: The Toronto study. Part III: Problems and complications encountered. J. Prosthet. Dent..

[3] Zarb G.A., Schmitt A. (1990). The longitudinal clinical effectiveness of osseointegrated dental implants: The Toronto Study. Part II: The prosthetic results. J. Prosthet. Dent..

[4] Zarb G.A., Schmitt A. (1990). The longitudinal clinical effectiveness of osseointegrated dental implants: The Toronto study. Part I: Surgical results. J. Prosthet. Dent..

[5] Schwarz M.S., Rothman S.L., Rhodes M.L., Chafetz N. (1987). Computed tomography: Part II. Preoperative assessment of the maxilla for endosseous implant surgery. Int. J. Oral Maxillofac. Implants.

[6] Schwarz M.S., Rothman S.L., Rhodes M.L., Chafetz N. (1987). Computed tomography: Part, I. Preoperative assessment of the mandible for endosseous implant surgery. Int. J. Oral Maxillofac. Implants.

[7] Bover-Ramos F., Vina-Almunia J., Cervera-Ballester J., Penarrocha-Diago M., Garcia-Mira B. (2018). Accuracy of Implant Placement with Computer-Guided Surgery: A Systematic Review and Meta-Analysis Comparing Cadaver, Clinical, and in Vitro Studies. Int. J. Oral Maxillofac. Implants.

[8] Cristache C.M., Gurbanescu S. (2017). Accuracy Evaluation of a Stereolithographic Surgical Template for Dental Implant Insertion Using 3D Superimposition Protocol. Int. J. Dent..

[9] van Steenberghe D., Glauser R., Blomback U., Andersson M., Schutyser F., Pettersson A., Wendelhag I. (2005). A computed tomographic scan-derived customized surgical template and fixed prosthesis for flapless surgery and immediate loading of implants in fully edentulous maxillae: A prospective multicenter study. Clin. Implant. Dent. Relat Res..

[10] Weiss R., Read-Fuller A. (2019). Cone Beam Computed Tomography in Oral and Maxillofacial Surgery: An Evidence-Based Review. Dent. J..

[11] Verstreken K., Van Cleynenbreugel J., Marchal G., Naert I., Suetens P., van Steenberghe D. (1996). Computer-assisted planning of oral implant surgery: A three-dimensional approach. Int. J. Oral Maxillofac. Implants.

[12] Vercruyssen M., Laleman I., Jacobs R., Quirynen M. (2015). Computer-supported implant planning and guided surgery: A narrative review. Clin. Oral Implants Res..

[13] Schneider D., Marquardt P., Zwahlen M., Jung R.E. (2009). A systematic review on the accuracy and the clinical outcome of computer-guided template-based implant dentistry. Clin. Oral Implants Res..

[14] D’Haese J., Ackhurst J., Wismeijer D., De Bruyn H., Tahmaseb A. (2017). Current state of the art of computer-guided implant surgery. Periodontol. 2000.

[15] Al Mortadi N., Eggbeer D., Lewis J., Williams R.J. (2012). CAD/CAM/AM applications in the manufacture of dental appliances. Am. J. Orthod Dentofacial Orthop..

[16] Davidowitz G., Kotick P.G. (2011). The use of CAD/CAM in dentistry. Dent. Clin. N. Am..

[17] Cordaro L., Torsello F., Ribeiro C.A. (2010). Transition from a failing dentition to a removable implant-supported prosthesis: A staged approach. Quintessence Int..

[18] Drew H.J., Alnassar T., Gluck K., Rynar J.E. (2012). Considerations for a staged approach in implant dentistry. Quintessence Int..

[19] el Attar M.S., el Shazly D., Osman S., el Domiati S., Salloum M.G. (1999). Study of the effect of using mini-transitional implants as temporary abutments in implant overdenture cases. Implant Dent..

[20] Gallucci G.O., Finelle G., Papadimitriou D.E., Lee S.J. (2015). Innovative approach to computer-guided surgery and fixed provisionalization assisted by screw-retained transitional implants. Int. J. Oral Maxillofac. Implants.

[21] Jivraj S., Reshad M., Chee W.W. (2008). Transitioning patients from teeth to implants utilizing fixed restorations. J. Calif. Dent. Assoc..

[22] Petrungaro P.S., Windmiller N. (2001). Using transitional implants during the healing phase of implant reconstruction. Gen. Dent..

[23] Cordaro L., Torsello F., Ercoli C., Gallucci G. (2007). Transition from failing dentition to a fixed implant-supported restoration: A staged approach. Int. J. Periodontics Restorative Dent..

[24] Greenstein G., Cavallaro J. (2008). Serial extraction protocol: Transitioning a hopeless dentition to a full-arch reconstruction. Compend. Contin. Educ. Dent..

[25] Wittneben J.G., Avdic M., Wright R.F., Radics A., Gallucci G.O., Weber H.P. (2009). Fixed mandibular and maxillary implant rehabilitation in a fully edentulous patient: A case report. Int. J. Periodontics Restorative Dent..

[26] Allen P.F., McMillan A.S. (2003). A review of the functional and psychosocial outcomes of edentulousness treated with complete replacement dentures. J. Can. Dent. Assoc..

[27] Cho S.C., Shetty S., Froum S., Elian N., Tarnow D. (2007). Fixed and removable provisional options for patients undergoing implant treatment. Compend. Contin. Educ. Dent..

[28] Lee S.J., Gallucci G.O. (2013). Digital vs. conventional implant impressions: Efficiency outcomes. Clin. Oral Implants Res..

[29] Bozini T., Petridis H., Garefis K., Garefis P. (2011). A meta-analysis of prosthodontic complication rates of implant-supported fixed dental prostheses in edentulous patients after an observation period of at least 5 years. Int. J. Oral Maxillofac. Implants.

[30] Stawarczyk B., Ozcan M., Schmutz F., Trottmann A., Roos M., Hammerle C.H. (2013). Two-body wear of monolithic, veneered and glazed zirconia and their corresponding enamel antagonists. Acta Odontol. Scand..

[31] Zhang Y., Lee J.J., Srikanth R., Lawn B.R. (2013). Edge chipping and flexural resistance of monolithic ceramics. Dent. Mater..

[32] Preis V., Weiser F., Handel G., Rosentritt M. (2013). Wear performance of monolithic dental ceramics with different surface treatments. Quintessence Int..

[33] Sripetchdanond J., Leevailoj C. (2014). Wear of human enamel opposing monolithic zirconia, glass ceramic, and composite resin: An in vitro study. J. Prosthet. Dent..

[34] Karakoca S., Yilmaz H. (2009). Influence of surface treatments on surface roughness, phase transformation, and biaxial flexural strength of Y-TZP ceramics. J. Biomed. Mater. Res. B Appl. Biomater..

[35] Preis V., Schmalzbauer M., Bougeard D., Schneider-Feyrer S., Rosentritt M. (2015). Surface properties of monolithic zirconia after dental adjustment treatments and in vitro wear simulation. J. Dent..

